# Analysis of Clinical Characteristics of Patients with Systemic Sclerosis and Gastric Antral Vascular Ectasia

**DOI:** 10.3390/jcm15093526

**Published:** 2026-05-05

**Authors:** Claudia Codina-Clavaguera, Luis Gerardo Alcala-Gonzalez, Laura Triginer-Gil, Alejandra Fernandez-Luque, Francisco-Alejandro Félix-Téllez, Maria Teresa Sanz-Martínez, Laura Viñas-Giménez, Janire Perurena-Prieto, Alfredo Guillen-Del-Castillo, Carmen P. Simeón-Aznar

**Affiliations:** 1 Systemic Autoimmune Diseases Unit, Internal Medicine Department, Vall d’Hebron University Hospital (HUVH), Vall d’Hebron Barcelona Hospital Campus, 08035 Barcelona, Spain; claudia.codina@vallhebron.cat (C.C.-C.); laura.triginer@hotmail.es (L.T.-G.); alejandra.fernandez@vallhebron.cat (A.F.-L.); carmenpilar.simeon@vallhebron.cat (C.P.S.-A.); 2 Department of Medicine, Universitat Autònoma de Barcelona, 08035 Barcelona, Spain; 3 Digestive System Research Unit, Department of Digestive Diseases, Vall d’Hebron University Hospital (HUVH), Vall d’Hebron Barcelona Hospital Campus, 08035 Barcelona, Spainftellezmd@gmail.com (F.-A.F.-T.); 4 Immunology Division, Vall d’Hebron University Hospital (HUVH), Vall d’Hebron Barcelona Hospital Campus, 08035 Barcelona, Spain; mariateresa.sanz@vallhebron.cat (M.T.S.-M.); laura.vinas@vallhebron.cat (L.V.-G.); janire.perurena@vallhebron.cat (J.P.-P.)

**Keywords:** Systemic Sclerosis, gastric antral vascular ectasia (GAVE)

## Abstract

**Background/Objectives**: Gastric antral vascular ectasia (GAVE) is a gastrointestinal manifestation associated with systemic sclerosis (SSc) that can lead to significant morbidity. This study aimed to characterise and compare the clinical profiles, laboratory findings, therapeutic approaches and survival outcomes of SSc patients with and without GAVE, based on data obtained during their first oesophagogastroduodenoscopy (EGD). **Methods**: A total of 269 patients who had undergone at least one EGD were selected. Twenty-seven were diagnosed with GAVE and compared with the remaining 242. **Results**: The overall prevalence of GAVE in SSc patients was 10%. Patients with GAVE had specific features such as a higher median age SSc onset (56.6 vs 48.0 years, *p* = 0.001), a higher prevalence of Barrett’s oesophagus (14.8% vs. 3.7%, *p* = 0.011), intestinal involvement (37% vs. 18.6%, *p* = 0.024) and a trend towards a lower prevalence of interstitial lung disease (25.9% vs. 45.0%, *p* = 0.057). A higher frequency of early or active Cutolo capillaroscopy pattern (84.6% vs. 62.4%, *p* = 0.025), greater frequency of anti-centromere antibodies (63.0% vs. 42.1%, *p* = 0.039) and a trend towards a lower proportion of anti-topoisomerase I (3.7% vs. 18.6%, *p* = 0.052) was also observed. No difference was found in the prevalence of anti-RNA polymerase III antibodies, survival or mortality. **Conclusions**: SSc patients with GAVE exhibit a distinct phenotype characterised by older age at disease onset, gastrointestinal involvement, anti-centromere antibodies and early or active capillaroscopic pattern, without differences in survival.

## 1. Introduction

Systemic sclerosis (SSc) is a systemic autoimmune disease characterised by endothelial damage, autoimmunity and fibrosis, processes that might involve skin and internal organs [1]. Gastric antral vascular ectasia (GAVE) was first described endoscopically in 1953 by Rieder et al. [2] as an erosive type of gastritis with marked veno-capillary ectasia in a patient who presented with chronic iron deficiency anaemia. In 1984, it was more accurately described as longitudinal antral folds converging on the pylorus, containing visible columns of tortuous red ectasic vessels, features which are now considered pathognomonic for GAVE diagnosis [3]. These endoscopic appearances resemble the stripes on a watermelon, hence the term “watermelon stomach” [4]. It is well known that liver cirrhosis is present in approximately 30% of GAVE patients, whilst the 60% of non-cirrhotic GAVE patients have an underlying autoimmune connective tissue disease, most commonly SSc [5,6,7]. Although its association with SSc is well-recognised, its exact pathogenesis remains unknown. Given the fact that the histological features seen in GAVE include the presence of hyperplasia of the lamina propria and abnormal vessels in the submucosa, it has been postulated that GAVE is more a vascular rather than a specific gastric manifestation of SSc, similar to pulmonary hypertension (PH) and scleroderma renal crisis (SRC) [8]. Other authors have linked GAVE with an autoimmune process, supporting this theory by the fact that GAVE has been associated with other autoimmune diseases in population studies [9]. Furthermore, several autoantibodies have been detected in patients with GAVE, including antinuclear antibodies (ANAs), with anti-centromere (ACA) and anti-RNA polymerase III (ARA) [8] the most reported ones. The epidemiology remains unclear and disparate, ranging from 0.6% in a Brazilian SSc cohort [10] to 1% in the EUSTAR network [11] and 10.6% in the Australian group [8]. Given there is no data published specifically in the Spanish population comparing cohorts, the objectives of our study were: first, to define the GAVE prevalence in SSc patients undergoing their first oesophagogastroduodenoscopy (EGD); secondly, to clarify the clinical manifestations that were associated with this complication, the previous treatments received, and its specific management. Lastly, we also aimed to evaluate its impact on patient survival.

## 2. Materials and Methods

### 2.1. Ethics

The study protocol was approved by the Ethics Committee for Clinical Research (PG(AG)4/2015). All participants provided written informed consent to participate in accordance with the principles outlined in the Declaration of Helsinki.

### 2.2. Study Design

This was an observational, retrospective, cohort study performed in a prospectively collected database of patients with SSc in the tertiary Vall d’Hebron University Hospital. All patients met the classification proposed by LeRoy and Medsger 2001 or the 2013 ACR/EULAR classification criteria [12,13]. The study protocol was approved by the Ethical Review Board of our institution (PG(AG)07/2015). All methods were performed in accordance with relevant guidelines and regulations, and patients gave written informed consent for the management of clinical data.

For the present study, only SSc patients selected by their first oesophagogastroduodenoscopy (EGD) were included in the analysis. The indications of upper endoscopy studies were dysphagia, severe GERD or anaemia. For the case–control analysis, we compared patients with and without GAVE as verified by endoscopic reports.

### 2.3. Data Collection

Demographic, clinical, immunological, and capillaroscopy data were collected as previously published [14,15,16,17]. Age at onset of disease was defined as the age of the patient at the first clinical symptom reported by the patient, including Raynaud’s phenomenon (RP). The age at onset of the first non-RP symptom was also included. The age at first EGD and the time from SSc onset until the EGD was performed were also collected. Patients were classified according to Leroy and Medsger’s cutaneous subsets [13] into limited cutaneous SSc (lcSSc), diffuse cutaneous SSc (dcSSc) or sine scleroderma SSc (ssSSc). Clinical manifestations of SSc were recollected with actual definitions and were recollected following the current definitions and according to our latest studies [16,18].

Gastrointestinal symptoms of GERD, such as dysphagia and heartburn, were collected at the time of upper endoscopy. The frequencies of other gastrointestinal (GI) manifestations were also compiled, not only those related to inflammation but also those regarding motility and hepatic involvement. Upper GI bleeding was encompassed as GAVE clinical manifestations, and digestive hypomotility was defined by findings on oesophageal manometry or oesophageal/intestinal barium radiography.

Antinuclear antibodies (ANAs) were identified by indirect immunofluorescence (IIF) assay using the Hep-2 cell line (INOVA, San Diego, CA, USA). SSc-specific autoantibodies were determined by commercial line blot assay (Systemic Sclerosis Profile Euroline ^®^ Blot test kit, Euroimmun, Lübeck, Germany). Data was collected on the treatment received, both the pharmacological treatment received prior to the first EGD, including calcium channel blockers, specific vasodilators, and immunosuppressants, and the treatment received for GAVE (endoscopic fulguration, iron supplementation, or blood product transfusions). Mortality was assessed and recorded in the registry by reviewing medical files.

### 2.4. Statistical Analysis

The median and interquartile range (IQR) were calculated for the quantitative variables, while the number and percentage were calculated for the qualitative variables. The Mann–Whitney U test was used to analyse the quantitative variables. The Chi-square test, Fisher’s exact test and odds ratio were performed for the comparative analysis of qualitative variables. Kaplan–Meier survival curves and the long Rank test were used to determine the survival between groups. Multivariate regression models adjusted by confounding variables have been used to avoid spurious associations. Tests were considered significant when the *p*-value was < 0.05. When a problem of complete separation occurred, we used Firth’s penalised logistic regression to obtain bias-reduced estimates.

## 3. Results

A total of 269 patients who had undergone at least one EGD during follow-up were selected. Demographics and clinical data at first EGD were taken into account for further analysis. Twenty-seven of them were diagnosed with GAVE at their first EGD, with the prevalence of GAVE in our cohort of 10.0%. From the whole cohort, 89.2% were females, and 21.6% were diffuse SSc (Table 1). The median (IQR) age at the disease onset was 45.2 years (29.9–60.5), and the median age at onset of the first non-RP symptom was 49.9 years (35.3–64.5). Concerning the age at first EGD, the median age was 57 years (43.1–70.9), and the median time to EGD from SSc onset was 8.6 years (3–18.4).

Patients with GAVE were older at SSc onset, considering the first non-Raynaud’s phenomenon (RP) symptom attributable to the disease (56.6 vs. 48.0 years, *p* = 0.001), but no differences were found in terms of gender, SSc subsets, or age at first EGD.

### 3.1. Gastrointestinal Characteristics

In the global cohort, 89.3% of patients fulfilled the 2013 ACR/EULAR criteria, and 96.7% of them had RP. No differences were found in terms of GERD symptoms, but the most prevalent in the overall cohort were heartburn (58%) and dysphagia (42%). Compared with SSc patients without GAVE, patients with GAVE had a higher prevalence of Barrett’s oesophagus (14.8% vs. 3.7%, *p* = 0.011) and intestinal involvement (37.0% vs. 18.6%, *p* = 0.024). In terms of other gastric manifestations, all patients with GAVE who underwent gastric motility studies were found to have delayed gastric emptying (2/2 100% vs. 5/7 71.4% *p* = 1.000) and also more chronic gastritis (40.7% vs. 21.1%, *p* = 0.021). Other gastrointestinal manifestations, like small intestinal bacterial overgrowth (22/70, 31.4%) and intestinal hypoperistalsis (50%), were observed in the global cohort without statistical differences between groups, although not all patients underwent the specific diagnostic gastrointestinal tests.

### 3.2. Other Clinical Characteristics

No differences were found concerning other vascular manifestations between GAVE and non-GAVE groups, with the prevalence of telangiectasia and digital ulcers in the global cohort being 76.6% and 46.1%, respectively.

In the overall cohort, 51.3% of patients had lung involvement, and no differences were observed between groups except a trend towards a lower prevalence of ILD in the GAVE group (25.9% vs. 45.0%, *p* = 0.057). Similarly, a numerically higher prevalence of PH (22.5% vs. 12.4%, *p* =0.155) and a tendency towards lower DLCO values during follow-up (47.5% vs. 59%, *p* = 0.053) were also observed in the GAVE group. Regarding cardiac involvement, the prevalence in the entire cohort was 68.8%, and no differences were observed between the two groups. No differences were also noted concerning musculoskeletal involvement or cancer prevalence (20.8%). None of the patients in the GAVE group developed scleroderma renal crisis.

### 3.3. Nailfold Capillaroscopy and Immunological Features

Significant differences between groups were observed in capillaroscopic findings, with patients diagnosed with GAVE exhibiting a higher prevalence of early or active pattern with a predominance of enlarged capillaries or giant capillaries (84.6% vs. 62.4%, *p* = 0.025), with a tendency to lower late capillaroscopy pattern (7.7% vs. 24.8%, *p* = 0.051).

Regarding the antibody profile, a higher frequency of ACA was observed in the GAVE patient group compared to those without GAVE (63% vs. 42.1%, *p* = 0.039). Additionally, there was a trend toward a lower frequency of ATA in the GAVE group (3.7% vs. 18.6%, *p* = 0.052), although this difference did not reach statistical significance. No differences were observed concerning the prevalence of anti-RNA polymerase III antibodies, with a global positivity of 10.2%.

### 3.4. Prior Therapies and GAVE Management

In relation to previous vasodilator treatment received (Table 2), calcium channel blockers were the most frequently used in the overall cohort (46.5%), with no significant differences observed between the two groups. Other vasodilator therapies in the global population of patients included endothelin receptor antagonists (ERAs) (8.9%), prostaglandins (5.6%), and phosphodiesterase type 5 inhibitors (PDE-5is) (5.6%).

In the overall cohort, 33.1% had received immunosuppressive therapy, and corticosteroids were commonly used (30.5%), followed by non-corticosteroid immunosuppressants (18.2%). Interestingly, patients with GAVE were treated less frequently with non-corticosteroid immunosuppressants (0% vs. 20.2%, *p* = 0.010). Given that none of the patients with GAVE had received non-corticosteroid immunosuppressants prior to EGD, a problem of complete separation occurred. Therefore, Firth’s penalised logistic regression was used to obtain bias-reduced estimates. In this model, adjusting for ILD and ACA, the use of non-corticosteroid immunosuppressants prior to endoscopy remained independently associated with lower odds of GAVE (OR 0.10, 95% CI 0.001–0.79, *p* = 0.024). In patients without GAVE, the most commonly used immunosuppressants were mycophenolate (8.3%), azathioprine (7.9%), cyclophosphamide (7.4%), methotrexate (4.5%), and calcineurin inhibitors (3.7%).

Regarding the specific management for GAVE, the main treatments used were oral iron supplementation (66.7%) and endoscopic fulguration (33.3%). Six of the nine patients who required fulguration needed it on more than one occasion during follow-up, with a median of two sessions (IQR 1-3.5).

### 3.5. Mortality and Survival Analysis

The mortality in our cohort was 33.8%, with no differences observed between the two groups (Table 3). The main SSc-related causes of death in GAVE patients were pulmonary arterial hypertension (PAH) (7.4%) and cardiovascular conditions (3.7%). However, the main SSc-related causes in non-GAVE patients were cardiovascular conditions (6.2%), PAH (5.8%), ILD (3.7%) and scleroderma renal crisis (2.9%). When considering non-SSc-related causes of death, cancer and hepatic involvement were present in both groups.

**Figure 1:**
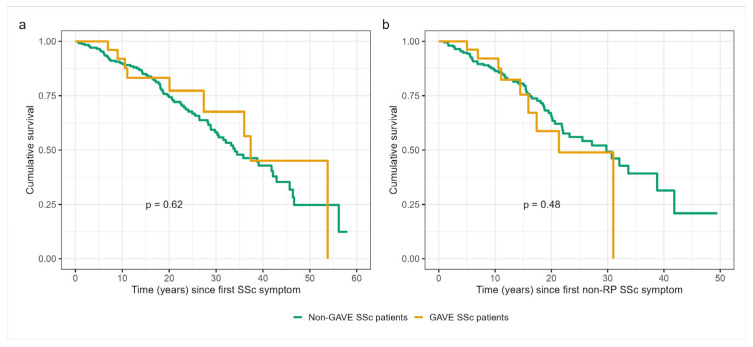
Kaplan–Meier survival curves comparing patients with and without GAVE. (**a**) Survival from the first systemic sclerosis (SSc) symptom and (**b**) survival from the first non-Raynaud’s phenomenon (non-RP) SSc symptom.

Survival analysis revealed no statistically significant differences between GAVE and non-GAVE subgroups of SSc patients, either from the onset of the first SSc symptom or from the first non-RP symptom (Figure 1). From the first SSc symptom, survival rates at 5, 10, 15, and 20 years were 100%, 100%, 92.0%, 83.2%, and 83.2% in GAVE patients and 96.6%, 89.6%, 85.0%, and 74.3% in non-GAVE patients, respectively (log-rank *p* = 0.60). When the analysis was performed from the first non-RP SSc symptom, survival rates at 5, 10, 15, and 20 years were 100%, 100%, 92.1%, 75.5%, and 58.7% in GAVE patients and 94.8%, 87.0%, 80.6%, and 65.8% in non-GAVE patients, respectively (log-rank *p* = 0.50).

## 4. Discussion

Our study identifies a distinct clinical phenotype in SSc patients with GAVE characterised by an older age at onset of the first non-RP symptom and a higher prevalence of Barrett’s oesophagus and intestinal involvement, with a higher frequency of enlarged capillaries or giant capillaries on capillaroscopy and ACAs in the immunology test. GAVE patients were less commonly treated with non-corticosteroid immunosuppressants prior to the endoscopic diagnosis, but no statistically significant differences were observed in survival rates.

A notable strength of our study is that it provides data on the prevalence of GAVE (10%) in a real-world Spanish cohort of patients with SSc, for whom endoscopy was performed based on clinical indications (dysphagia, severe GERD, or anaemia). This differentiates the present study from others, such as the SCOT trial [5], in which all early dcSSc patients underwent endoscopy regardless of GI symptoms, a fact that might have led to overestimation of the incidence of GAVE. Our findings are consistent with those of the Australian cohort [8], where the indications for endoscopy were similar to our study (occult or acute GI bleeding, delayed gastric emptying on nuclear studies, refractory symptoms, GERD, or dysphagia). Furthermore, our results support the notion that the wide range of GAVE prevalence reported in the literature (1–22%) may be largely attributable to differences in the indications for endoscopy (asymptomatic versus symptomatic GI disease) rather than differences in clinical characteristics [5,8,19]. Only a systematic endoscopic evaluation, which was beyond the scope of our study, could give the real prevalence of GAVE in patients with SSc. Moreover, GAVE can be challenging to identify during endoscopy, which may result in its misdiagnosis. This finding was further emphasised by Kitiyakara et al., who identified 6/128 patients (4.7%) with GAVE that was not detected by routine endoscopy [20]. Nonetheless, the utilisation of endoscopic videocapsules remains a subject of debate within the domain of SSc, primarily due to concerns regarding safety or even cost-effectiveness [20].

Consistent with previous literature, patients with GAVE in our cohort were older at SSc disease onset [2]. However, they had a lower prevalence of the diffuse SSc subtype (18.5%), in contrast to the EUSTAR cohort [5] and also the Australian cohort [8], which reached 49% and 35.3% for diffuse SSc, respectively. This is congruent with the antibody profile of our GAVE population, where a clearly higher frequency of ACAs was observed, but also a lower proportion of anti-RNA polymerase III.

As the exact pathogenesis of GAVE in systemic sclerosis is unknown and can be classified either as a gastric or vascular manifestation, or a combination of both [9,21], we believe that these findings bring to the forefront the need to further investigate the underlying mechanisms involved. Notably, the increased frequency of an early or active capillaroscopic pattern in almost 85% of the SSc patients with GAVE suggests that this manifestation might take part in a vascular phenotype [11]. The tendency towards lower DLCO values and a higher numerical proportion of PH in this population could be interpreted as possible support for this vascular hypothesis rather than a purely gastric manifestation. Similarly, although the EUSTAR group did not find a clear association between ILD and GAVE, some observations of reduced DLCO/AV (a predictor for PH) values have been reported [11], which may warrant further investigation.

In this regard, studies have been published linking the presence of GAVE with biomarkers of angiogenesis like angiopoietin 2 (Ang-2), which also supports the idea that it might be associated with a disbalance in vascular homeostasis [22]. A shared pathogenetic mechanism with hereditary haemorrhagic teleangiectasia, an autosomal dominant disorder of vasculature affecting the gut in up to 30% of cases, has also been proposed in other studies [23,24]. We consider that the absence of a correlation between GAVE and teleangiectasias in our study may be explained by the fact that teleangiectasias are a very common feature in our cohort (76.6% of the overall population) rather than a lack of correlation with a vascular phenotype, as we mentioned previously.

Regarding gastrointestinal involvement, a significant association was identified between GAVE, Barrett’s oesophagus and intestinal involvement, consistent with findings from the Australian cohort [11], which also reported a correlation between GAVE and intestinal dysmotility. These findings highlight the need for more precise studies of gastrointestinal tract motility, which could help characterise gastric motility and determine whether the observed correlations with both Barrett’s oesophagus and intestinal dysmotility are related to alterations in gastric motility that may lead to gastroparesis, increased reflux, and the subsequent development of Barrett’s oesophagus. The fact that the two patients with GAVE in whom gastric motility was assessed presented with delayed gastric emptying reinforces this hypothesis and emphasises the need to characterise the motility of the entire gastrointestinal tract in patients with scleroderma to correlate it with clinical and histological findings, given that venous ectasia may result from altered motility.

Regarding SSc-specific autoantibodies, GAVE patients in our cohort had a trend towards a lower proportion of ATA antibodies (3.7% vs. 18.6%, *p* = 0.052), consistent with prior literature suggesting a possible association. The SCOT trial identified a negative association between GAVE and ATA, and other studies have similarly suggested that ATA-positive patients have a low or negligible risk of developing GAVE. In contrast, GAVE has been reported in up to 25% of patients with anti-RNA polymerase III antibodies, a prevalence notably higher than in the general SSc population [25]. Interestingly, the predominance of anti-RNA polymerase III in GAVE patients in our cohort differs from that reported in other studies, where its presence has even been identified as an independent associated risk factor, with an OR 4.62 (1.2–21.12) in the EUSTAR group and OR 10.98 (2.97–40.63) in the Serling-Boyd et al. cohort [11,18,26].

We identified that GAVE patients had been treated less frequently with non-glucocorticoid immunosuppression prior to diagnosis, but no differences were observed in the rest of the treatments received. This finding could be initially interpreted as being related to the specific phenotype of GAVE patients in our cohort, with a predominantly limited SSc subtype and a higher prevalence of ACA. As is well known, patients with limited SSc are less likely to receive intensive immunosuppressive therapy, including non-glucocorticoid immunosuppression. Nevertheless, after adjusting for ILD and ACA, prior use of non-corticosteroid immunosuppressive agents before endoscopy remained independently associated with a lower prevalence of GAVE (OR 0.10, 95% CI 0.001–0.79, *p* = 0.024), pointing to the possibility of an underlying immunological mechanism, as suggested in previous literature [8]. In line with this hypothesis, several studies and case series have suggested a potential therapeutic effect of cyclophosphamide on GAVE when administered for other indications, such as ILD or progressive skin involvement [27,28]. Other reports have described successful use of cyclophosphamide in refractory GAVE cases [29,30]. In this setting, a recent case report has shown positive results with tocilizumab in a patient with refractory GAVE to all endoscopic treatments [24]. Taken together, these findings point to the hypothesis that the underlying pathophysiology of GAVE involves an inflammatory process affecting the mucosal and submucosal layers prior to the development of vascular dilatation, a process that could potentially be prevented or modulated by immunosuppressive therapies. The higher prevalence of chronic gastritis in patients with GAVE found in our study (40.7% vs. 21.1%, *p* = 0.021) may support this hypothesis; however, this should be interpreted cautiously, and further studies are needed to clarify whether this represents an initial, potentially treatable inflammatory process. Further studies in this direction could provide insights into the relationship between these two phenomena and their potential reversibility with immunosuppressive treatment.

In terms of GAVE management, patients in our cohort were treated with iron and fulgurations according to the expert recommendations [31], and most of them needed the endoscopic procedure more than once during follow-up. Since endoscopic fulguration is an invasive intervention with associated risks and potential complications, the development of effective and durable non-invasive therapies, including pharmacological treatments, is presented as a challenge [32].

Although patients with GAVE in our cohort did not show differences in terms of survival and mortality compared with those without GAVE, morbidity was notorious, as they had to undergo repeated endoscopies, intravenous treatments and transfusions. A condition such as GAVE may substantially contribute to the baseline morbidity already present in patients with SSc [33,34,35] and gastrointestinal involvement, as it not only increases the likelihood of severe complications but also implies the need for treatments that are frequently repeated over time and are invasive, with an inherent risk of procedural complications.

Although the present study is based on a substantial number of systemic sclerosis patients undergoing EGD at a single centre, several limitations should be acknowledged. First, as our analysis was restricted to data from the first EGD, cases of GAVE that developed during subsequent follow-up may not have been captured, so the real prevalence may be underestimated. In addition, the fact that this study was conducted in a high-complexity referral centre with a specialised gastrointestinal bleeding unit may have led to an over-representation of more severe cases requiring a greater number of therapeutic and diagnostic interventions. Nevertheless, we believe that the trends and conclusions obtained in our work can be extrapolated to other cohorts, especially those without a high prevalence of dcSSc or anti-RNA polymerase III antibodies.

## 5. Conclusions

GAVE is an under-recognised manifestation of systemic sclerosis that should be effectively managed and treated when properly diagnosed. The main limitations in current knowledge lie in its etiopathogenesis (whether vascular, immune-mediated, or intrinsically gastric) and its associations with autoantibodies, capillaroscopic patterns, and systemic sclerosis phenotypes. Recognising these associations will not only facilitate diagnosis and treatment but also guide accurate screening strategies to prevent the morbidity caused by this condition. The association of GAVE with vascular phenotype, the high prevalence of ACA, and low prior immunosuppressive therapy should alert us to the possibility of its presence and guide us to perform additional tests for its diagnosis, especially in patients with older age at onset of the first non-RP symptom, a higher prevalence of Barrett’s oesophagus and intestinal involvement, higher frequency of enlarged capillaries on capillaroscopy and ACA-positive antibodies. Future research should focus on developing non-invasive diagnostic tools and on defining the optimal timing for implementing available screening measures in order to ensure earlier detection and specific therapy to improve the quality of life and clinical outcomes.

## Figures and Tables

**Table 1:** Descriptive analysis.

	Overall (n = 269)	GAVE SSc Patients (n = 27)	Non-GAVE SSc Patients (n = 242)	*p* Value
**Female gender, n (%)**	240 (89.2%)	23 (85.2%)	217 (89.7%)	0.509
**SSc subsets**				
Diffuse cutaneous SSc, n (%)	58 (21.6%)	5 (18.5%)	53 (21.9%)	0.685
Limited cutaneous SSc, n (%)	171 (63.6%)	19 (70.4%)	152 (62.8%)	0.729
*Sine scleroderma* SSc, n (%)	40 (14,9%)	3 (11.1%)	37 (15.3%)	0.720
**Age at disease onset, median (IQR)**	45.2 (29.9–60.5)	49.1 (30.7–67.5)	44.8 (29.9–59.7)	0.248
**Age at onset of first non-RP symptom, median (IQR)**	49.9 (35.3–64.5)	56.6 (41.2–72)	48.9 (34.6–63.2)	0.011
**Age at first EGD**	57 (43.1–70.9)	61.7 (47.6–75.8)	56.5 (42.7–70.3)	0.067
**Time to EGD from SSc onset, years (IQR)**	8.6 (3–18.4)	9.5 (2–20.4)	8.5 (3–17.8)	0.878
**2013 ACR/EULAR criteria, n (%)**	241 (89.6%)	25 (92.6%)	216 (89.3%)	0.590
**Comorbidities**				
Current smoker, n (%)	20 (7.4%)	0 (0.0%)	20 (8.3%)	0.121
HTA, n (%)	107 (39.8%)	14 (51.9%)	93 (38.4%)	0.177
Dyslipidemia, n (%)	58 (21.6%)	6 (22.2%)	52 (21.5%)	0.930
Diabetes, n (%)	15 (5.6%)	3 (11.1%)	12 (5.0%)	0.186
**Vascular manifestations:**				
**Raynaud’s phenomenon, n (%)**	260 (96.7%)	26 (96.3%)	234 (96.7%)	0.913
**Telangiectasias, n (%)**	206 (76.6%)	24 (88.9%)	182 (75.2%)	0.111
**Digital ulcers, n (%)**	124 (46.1%)	14 (51.9%)	110 (45.5%)	0.527
**Gastrointestinal manifestations, n (%)**				
Heartburn, n (%)	156 (58.0%)	16 (59.3%)	140 (57.9%)	0.888
Dysphagia, n (%)	113 (42.0%)	7 (25.9%)	106 (43.8%)	0.074
**Gastrointestinal involvement, n (%)**	248 (92.2%)	27 (100.0%)	221 (91.3%)	0.111
Oesophageal involvement, n (%)	229 (85.1%)	22 (81.5%)	207 (85.5%)	0.574
Oesophageal hypoperistalsis, n (%)	174 (86.6%)	15 (83.3%)	159 (86.9%)	0.673
Oesophagitis, n (%)	84 (31.2%)	12 (44.4%)	72 (29.8%)	0.118
Barrett’s oesophagus, n (%)	13 (4.8%)	4 (14.8%)	9 (3.7%)	0.011
Gastric involvement, n (%)	87 (32.3%)	27 (100.0%)	60 (24.8%)	<0.001
Delayed gastric emptying	7/9 (77.8%)	2/2 (100%)	5/7 (71.4%)	1.000
Chronic gastritis	62 (23%)	11 (40.7%)	51 (21.1%)	0.021
Intestinal involvement, n (%)	55 (20.4%)	10 (37.0%)	45 (18.6%)	0.024
Intestinal hypoperistalsis, n (%) (n = 16)	8/16 (50.0%)	1/2(50.0%)	7/14 (50.0%)	1.000
Small intestinal bacterial overgrowth, n (%)	22 (31.4%)	3 (33.3%)	19 (31.1%)	0.895
Hepatic involvement, n (%)	34 (12.6%)	6 (22.2%)	28 (11.6%)	0.114
**Lung involvement**	138 (51.3%)	12 (44.4%)	126 (52.1%)	0.452
Interstitial lung disease, n (%)	116 (43.1%)	7 (25.9%)	109 (45%)	0.057
Pulmonary arterial hypertension, n (%)	36 (13.4%)	6 (22.2%)	30 (12.4%)	0.155
**PFTs**				
**Baseline PFTs, median (IQR)**				
FVC, %, median (IQR)	81.3 (62–100.6)	82.7 (69–96.4)	81.1 (61.2–101)	0.595
DLCO, %, median (IQR)	63.3 (41.9–84.7)	58 (44.9–71.1)	63.5 (41.7–85.3)	0.618
**Cardiac involvement, n (%)**	185 (68.8%)	20 (74.1%)	165 (68.2%)	0.531
**Renal involvement**				
Scleroderma renal crisis, n (%)	8 (3.0%)	0 (0.0%)	8 (3.3%)	0.337
**Musculoskeletal involvement, n (%)**				
Arthralgias, n (%)	147 (54.6%)	12 (44.4%)	135 (55.8%)	0.262
Arthritis, n (%)	64 (23.8%)	3 (11.1%)	61 (25.2%)	0.103
Myositis, n (%)	21 (7.8%)	0 (0.0%)	21 (8.7%)	0.111
Contractures, n (%)	18 (6.7%)	2 (7.4%)	16 (6.6%)	0.875
Tendon friction rubs, n (%)	7 (2.6%)	0 (0.0%)	7 (2.9%)	0.371
Calcinosis, n (%)	65 (24.2%)	5 (18.5%)	60 (24.8%)	0.470
**Cancer**				
Total cancer, n (%)	56 (20.8%)	7 (25.9%)	49 (20.2%)	0.491
**Capillaroscopic findings, n (%)**				
Early or active capillaroscopy pattern, n (%)	153 (64.8%)	22 (84.6%)	131 (62.4%)	0.025
Late capillaroscopy pattern, n (%)	54 (19.3%)	2 (7.7%)	52 (24.8%)	0.051
**Antibody profile**				
ANA positivity, n (%)	257 (95.5%)	26 (96.3%)	231 (95.5%)	0.841
Anti-centromere, n (%)	119 (44.2%)	17 (63.0%)	102 (42.1%)	0.039
Anti-topo I, n (%)	45 (17.0%)	1 (3.7%)	44 (18.6%)	0.052
Anti-RNA polymerase III, n (%)	17 (10.2%)	3 (15.0%)	14 (9.5%)	0.447
Anti-PM/Scl, n (%)	12 (6.0%)	1 (4.0%)	11 (6.2%)	0.657
Anti-U1-RNP, n (%)	8 (3.1%)	0 (0.0%)	8 (3.5%)	0.322
Anti-Th-To, n (%)	3 (1.8%)	0 (0.0%)	3 (2.1%)	0.516
Anti-Ku, n (%)	3 (1.6%)	0 (0.0%)	3 (1.8%)	0.497
Anti-U3RNP, n (%)	2 (1.2%)	0 (0.0%)	2 (1.4%)	0.597

**Table 2:** Treatments.

	Overall (n = 269)	GAVE SSc Patients (n = 27)	Non-GAVE SSc Patients (n = 242)	*p* Value
**Treatment prior to GAVE diagnosis or EGD date**				
**Vasodilators**				
Calcium channel blockers, n (%)	125 (46.5%)	13 (48.1%)	112 (46.3%)	0.854
**Specific vasodilators**				
Endothelin receptor antagonists, n (%)	24 (8.9%)	3 (11.1%)	21 (8.7%)	0.674
Prostaglandins, n (%)	15 (5.6%)	1 (3.7%)	14 (5.8%)	0.655
Phosphodiesterase 5 inhibitors, n (%)	15 (5.6%)	1 (3.7%)	14 (5.8%)	0.655
**Immunosuppressants, n (%)**	89 (33.1%)	5 (18.5%)	84 (34.7%)	0.090
**Corticosteroids, n (%)**	82 (30.5%)	5 (18.5%)	77 (31.8%)	0.154
**Non-corticosteroid immunosuppressants, n (%)**	49 (18.2%)	0 (0.0%)	49 (20.2%)	0.010
Calcineurin inhibitors, n (%)	9 (3.3%)	0 (0.0%)	9 (3.7%)	0.308
Mycophenolate acid, n (%)	20 (7.4%)	0 (0.0%)	20 (8.3%)	0.121
Azathioprine, n (%)	19 (7.1%)	0 (0.0%)	19 (7.9%)	0.131
Cyclophosphamide, n (%)	18 (6.7%)	0 (0.0%)	18 (7.4%)	0.142
Methotrexate, n (%)	11 (4.1%)	0 (0.0%)	11 (4.5%)	0.258
Rituximab, n (%)	5 (1.9%)	0 (0.0%)	5 (2.1%)	0.451
**Treatment of GAVE**				
Iron treatment, n (%)	69 (25.7%)	18 (66.7%)	51 (21.1%)	<0.001
Fulguration, n (%)	9 (3.3%)	9 (33.3%)	0 (0.0%)	<0.001

**Table 3:** Mortality.

	Overall (n = 269)	GAVE SSc Patients (n = 27)	Non-GAVE SSc Patients (n = 242)	*p* Value
**Death, n (%)**	91 (33.8%)	9 (33.3%)	82 (33.9%)	0.954
** SSc-related causes of death:**				
Cardiovascular, n (%)	16 (5.9%)	1 (3.7%)	15 (6.2%)	0.484
PAH, n (%)	16 (5.9%)	2 (7.4%)	14 (5.8%)	0.484
ILD, n (%)	9 (3.3%)	0 (0.0%)	9 (3.7%)	0.484
Scleroderma renal crisis, n (%)	7 (2.6%)	0 (0.0%)	7 (2.9%)	0.484
Infection, n (%)	3 (1.1%)	0 (0.0%)	3 (1.2%)	0.484
Gastrointestinal, n (%)	2 (0.7%)	0 (0.0%)	2 (0.8%)	0.484
** Non-SSc-related causes of death:**				
Cancer, n (%)	15 (5.6%)	2 (7.4%)	13 (5.4%)	0.484
Hepatic involvement, n (%)	5 (1.9%)	2 (7.4%)	3 (1.2%)	0.484
Other, n (%)	13 (4.8%)	2 (7.4%)	11 (4.5%)	0.484

## Data Availability

The original contributions presented in the study are included in the article; further inquiries can be directed to the corresponding author.

## Abbreviations

The following abbreviations are used in this manuscript:

GAVE	Gastric Antral Vascular Ectasia
PAH	Pulmonary Arterial Hypertension
ILD	Interstitial Lung Disease
EGD	Oesophagogastroduodenoscopy

## References

[1] Volkmann E.R., Andréasson K., Smith V. (2023). Systemic sclerosis. Lancet.

[2] Rider J.A., Klotz A.P., Kirsner J.B. (1953). Gastritis with veno-capillary ectasia as a source of massive gastric hemorrhage. Gastroenterology.

[3] Jabbari M., Cherry R., Lough J.O., Daly D.S., Kinnear D.G., Goresky C.A. (1984). Gastric antral vascular ectasia: The watermelon stomach. Gastroenterology.

[4] Burak K.W. (2001). Portal hypertensive gastropathy and gastric antral vascular ectasia (GAVE) syndrome. Gut.

[5] Hung E.W., Mayes M.D., Sharif R., Assassi S., Machicao V.I., Hosing C., St. Clair E.W., Furst D.E., Khanna D., Forman S. (2013). Gastric Antral Vascular Ectasia and Its Clinical Correlates in Patients with Early Diffuse Systemic Sclerosis in the SCOT Trial. J. Rheumatol..

[6] Gostout C.J., Carpenter H.A. (1995). Portal hypertensive vasculopathy and mucosal vascular ectasias. Gastroenterology.

[7] Anderton R.M., Zhu A., Ayla A., Chavez M., Mayer A., Koym K., Guillen-Del-Castillo A., Alcala-Gonzalez L.G., Hughes M., McMahan Z.H. (2026). Gastric disease in systemic sclerosis: Spectrum, challenges, and insights from a systematic literature review. Semin. Arthritis Rheum..

[8] Morrisroe K., Hansen D., Stevens W., Sahhar J., Ngian G.-S., Hill C., Roddy J., Walker J., Proudman S., Nikpour M. (2022). Gastric antral vascular ectasia in systemic sclerosis: A study of its epidemiology, disease characteristics and impact on survival. Arthritis Res. Ther..

[9] Parrado R.H., Lemus H.N., Coral-Alvarado P.X., Quintana López G. (2015). Gastric Antral Vascular Ectasia in Systemic Sclerosis: Current Concepts. Int. J. Rheumatol..

[10] Assad A.P.L., Farias R., Gaspari C.N., Da Silva H.C., Andrade D.C.O., Sampaio-Barros P.D. (2020). Diagnosis and Management of Gastric Antral Vascular Ectasia: Experience in a Large Single Cohort of Patients with Systemic Sclerosis. JCR J. Clin. Rheumatol..

[11] Ghrénassia E., Avouac J., Khanna D., Derk C.T., Distler O., Suliman Y.A., Airo P., Carreira P.E., Foti R., Granel B. (2014). Prevalence, Correlates and Outcomes of Gastric Antral Vascular Ectasia in Systemic Sclerosis: A EUSTAR Case-control Study. J. Rheumatol..

[12] Van Den Hoogen F., Khanna D., Fransen J., Johnson S.R., Baron M., Tyndall A., Matucci-Cerinic M., Naden R.P., Medsger T.A., Carreira P.E. (2013). 2013 classification criteria for systemic sclerosis: An American college of rheumatology/European league against rheumatism collaborative initiative. Ann. Rheum. Dis..

[13] LeRoy E.C., Medsger T.A. (2001). Criteria for the classification of early systemic sclerosis. J. Rheumatol..

[14] Felix-Tellez F.A., Guillen-del-Castillo A., Pedroza C., Aguilar A., Barber C., Malagelada C., Polo-Figueras L., Triginer L., Codina-Clavaguera C., Hughes M. (2026). Delayed gastric emptying identifies a high-risk clinical subgroup in patients with systemic sclerosis. Rheumatology.

[15] Félix-Téllez F.A., Guillén-Del-Castillo A., Serra Pueyo J., Aguilar A., Barber C., Codina C., Marín-García A., Malagelada C., Simeon-Aznar C.P., Alcalá-González L.G. (2025). Fecal Incontinence in Systemic Sclerosis: Prevalence, Clinical Correlates, and Impact on Quality of Life. Rev. Esp. Enfermedades Dig..

[16] Alcala-Gonzalez L.G., Guillen-del-Castillo A., Aguilar A., Barber C., Malagelada C., Polo Figueras L., Triginer L., Codina-Clavaguera C., Hughes M., Serra J. (2025). Oesophageal dysmotility patterns are associated with distinct clinical phenotypes and prognosis in patients with systemic sclerosis. Rheumatology.

[17] Alcala-Gonzalez L.G., Guillen-del-Castillo A., Aguilar Cayuelas A., Barber Caselles C., Codina-Clavaguera C., Marin García A., Serra J., Malagelada C., Simeón-Aznar C.P. (2025). Gastrointestinal dysmotility is associated with proton pump inhibitor refractory oesophagitis in patients with systemic sclerosis. Rheumatology.

[18] Callejas-Moraga E.L., Guillén-Del-Castillo A., Marín-Sánchez A.M., Roca-Herrera M., Balada E., Tolosa-Vilella C., Fonollosa-Pla V., Simeón-Aznar C.P. (2019). Clinical features of systemic sclerosis patients with anti-RNA polymerase III antibody in a single centre in Spain. Clin. Exp. Rheumatol..

[19] Marie I., Ducrotte P., Antonietti M., Herve S., Levesque H. (2008). Watermelon stomach in systemic sclerosis: Its incidence and management. Aliment. Pharmacol. Ther..

[20] Kitiyakara T., Selby W. (2005). Non-small-bowel lesions detected by capsule endoscopy in patients with obscure GI bleeding. Gastrointest. Endosc..

[21] Alcala-Gonzalez L.G., Hinchcliff M., McMahan Z.H. (2025). Gastrointestinal manifestations of systemic sclerosis: Current approaches and emerging therapies. Curr. Opin. Rheumatol..

[22] Douglas A.R., Holleran G., Smith S.M., McNamara D. (2020). Shared changes in angiogenic factors across gastrointestinal vascular conditions: A pilot study. World J. Gastrointest. Pharmacol. Ther..

[23] Zarka J., Jeong K., Yabes J.G., Ragni M.V. (2023). Prevalence and risk factors for bleeding in hereditary hemorrhagic telangiectasia: A National Inpatient Sample study. Blood Adv..

[24] Rodolfi S., Denton C.P., Ong V.H. (2025). Systemic sclerosis-associated severe gastric antral vascular ectasia treated with tocilizumab: A case report and review of the literature. J. Scleroderma Relat. Disord..

[25] Cavazzana I., Angela C., Paolo A., Stefania Z., Angela T., Franco F. (2009). Anti-RNA polymerase III antibodies: A marker of systemic sclerosis with rapid onset and skin thickening progression. Autoimmun. Rev..

[26] Serling-Boyd N., Chung M.P.S., Li S., Becker L., Fernandez-Becker N., Clarke J., Fiorentino D., Chung L. (2020). Gastric antral vascular ectasia in systemic sclerosis: Association with anti-RNA polymerase III and negative anti-nuclear antibodies. Semin. Arthritis Rheum..

[27] Lorenzi A.R., Johnson A.H., Davies G., Gough A. (2001). Gastric antral vascular ectasia in systemic sclerosis: Complete resolution with methylprednisolone and cyclophosphamide. Ann. Rheum. Dis..

[28] Schulz S.W., O’Brien M., Maqsood M., Sandorfi N., Galdo F.D., Jimenez S.A. (2009). Improvement of Severe Systemic Sclerosis-associated Gastric Antral Vascular Ectasia Following Immunosuppressive Treatment with Intravenous Cyclophosphamide. J. Rheumatol..

[29] Matsumoto Y., Hayashi H., Tahara K., Yasuda T., Tsubouchi S., Yamamoto Y., Mizuuchi T., Mori H., Sawada T. (2019). Intravenous Cyclophosphamide for Gastric Antral Vascular Ectasia Associated with Systemic Sclerosis Refractory to Endoscopic Treatment: A Case Report and Review of the Pertinent Literature. Intern. Med..

[30] Papachristos D.A., Nikpour M., Hair C., Stevens W.M. (2015). Intravenous cyclophosphamide as a therapeutic option for severe refractory gastric antral vascular ectasia in systemic sclerosis. Intern. Med. J..

[31] Selinger C.P., Ang Y.S. (2008). Gastric Antral Vascular Ectasia (GAVE): An Update on Clinical Presentation, Pathophysiology and Treatment. Digestion.

[32] Peng M., Guo X., Yi F., Romeiro F.G., Mancuso A., Qi X. (2021). Pharmacotherapy for the Treatment of Gastric Antral Vascular Ectasia: A Narrative Review. Adv. Ther..

[33] Volkmann E.R., McMahan Z. (2022). Gastrointestinal involvement in systemic sclerosis: Pathogenesis, assessment and treatment. Curr. Opin. Rheumatol..

[34] Alcala-Gonzalez L.G., Burton-Murray H., Atkins M., Guillen-Del-Castillo A., Malagelada C., Hughes M., McMahan Z.H., Simeón-Aznar C.P. (2025). Avoidant or Restrictive Food Intake Disorder Symptoms in Adults with Systemic Sclerosis: A Nationwide Study in Spain. Arthritis Care Res..

[35] Alcala-Gonzalez L.G., Guillen-Del-Castillo A., Aguilar A., Barber C., Codina C., Marin Garcia A., Malagelada C., Simeon-Aznar C.P. (2024). Impact of gastrointestinal symptoms and psychological distress on quality of life in systemic sclerosis: A cross-sectional study. BMJ Open.

